# Supplementary material for: mRNA Covid-19 vaccines in pregnancy: A systematic review
Source: PLoS One. 2022 Feb 2;17(2):e0261350. doi: 10.1371/journal.pone.0261350 (PMC8809595; doi:10.1371/journal.pone.0261350)
Supplement: S1 File — (DOCX) [file pone.0261350.s002.docx]

**S1.** **Literature Search**

- Keywords

#1 (SARS-CoV-2) OR (COVID-19)

#2 (Pregnancy) OR (Pregnant)

#3 (Vaccine) OR (Vaccination)

- Search Results

| **Database** | **Keywords** | **Search Result** | **Search-time** |
| --- | --- | --- | --- |
| MEDLINE | #1 AND #2 AND #3 | 275 | June 20, 2021 |
| ScienceDirect | #1 AND #2 AND #3 | 1787 | June 20, 2021 |
| Proquest | #1 AND #2 AND #3 | 1891 | June 20, 2021 |
| Springer | #1 AND #2 AND #3 | 94 | June 21, 2021 |
| Cochrane Library | #1 AND #2 AND #3 | 65 | June 20, 2021 |
| medRxiv | #1 AND #2 AND #3 | 168 | June 22, 2021 |
| bioRxiv | #1 AND #2 AND #3 | 22 | June 22, 2021 |
| Clinicaltrial.gov | #1 AND #2 AND #3 | 9 | June 20, 2021 |
| Manual searching | - | 1 | July 13, 2021 |
