# Supplementary material for: mRNA Covid-19 vaccines in pregnancy: A systematic review
Source: PLoS One. 2022 Feb 2;17(2):e0261350. doi: 10.1371/journal.pone.0261350 (PMC8809595; doi:10.1371/journal.pone.0261350)
Supplement: S1 Table — (DOCX) [file pone.0261350.s003.docx]

**S1 Table. Newcastle-Ottawa Scale (NOS) quality assessment of each included cohort study**

| Study | **Selection** | | | | **Comparability** | | **Outcome** | | |  |
| --- | --- | --- | --- | --- | --- | --- | --- | --- | --- | --- |
|  | Representativeness of exposed cohort | Selection of nonexposed cohort | Ascertainment of exposure | Demonstration that outcome of interest was not present at start of study | Adjust for the most important risk factors | Adjust for other risk factors | Assessment of outcome | Follow-up length | Loss to follow-up rate | Total quality score |
| Shimabukuro et al., 2021 | 🟊 | 🟊 | - | - | 🟊 | 🟊 | 🟊 | 🟊 | 🟊 | 7 |
| Gray et al., 2021 | 🟊 | 🟊 | 🟊 | - | 🟊 | 🟊 | 🟊 | 🟊 | 🟊 | 8 |
| Collier et al., 2021 | - | 🟊 | 🟊 | - | 🟊 | 🟊 | 🟊 | 🟊 | 🟊 | 7 |
| Shanes et al., 2021 | 🟊 | 🟊 | 🟊 | 🟊 | 🟊 | 🟊 | 🟊 | 🟊 | 🟊 | 9 |
| Prabhu et al., 2021 | - | - | 🟊 | 🟊 | 🟊 | 🟊 | 🟊 | 🟊 | 🟊 | 7 |
| Rottenstreich et al., 2021 | 🟊 | - | 🟊 | - | 🟊 | 🟊 | 🟊 | 🟊 | 🟊 | 7 |
| Theiler et al., 2021 | 🟊 | 🟊 | 🟊 | - | 🟊 | 🟊 | 🟊 | 🟊 | 🟊 | 8 |
| Beharier et al., 2021 | 🟊 | 🟊 | 🟊 | 🟊 | 🟊 | 🟊 | 🟊 | 🟊 | 🟊 | 9 |
| Goldshtein et al., 2021 | 🟊 | 🟊 | 🟊 | 🟊 | 🟊 | 🟊 | 🟊 | 🟊 | 🟊 | 9 |
