# Supplementary material for: mRNA Covid-19 vaccines in pregnancy: A systematic review
Source: PLoS One. 2022 Feb 2;17(2):e0261350. doi: 10.1371/journal.pone.0261350 (PMC8809595; doi:10.1371/journal.pone.0261350)
Supplement: S2 Table — (DOCX) [file pone.0261350.s004.docx]

**S2 Table. Joanna Briggs Institute (JBI) critical appraisal for case series study**

| No | Checklist questions | Mithal et al., 2021 |
| --- | --- | --- |
| 1. | Were there clear criteria for inclusion in the case series? | Yes |
| 2. | Was the condition measured in a standard, reliable way for all participants included in the case series? | Yes |
| 3. | Were valid methods used for identification of the condition for all participants included in the case series? | Yes |
| 4. | Did the case series have consecutive inclusion of participants? | Yes |
| 5. | Did the case series have complete inclusion of participants? | Yes |
| 6. | Was there clear reporting of the demographics of the participants in the study? | Yes |
| 7. | Was there clear reporting of clinical information of the participants? | Yes |
| 8. | Were the outcomes or follow up results of cases clearly reported? | Yes |
| 9. | Was there clear reporting of the presenting site(s)/clinic(s) demographic information? | Yes |
| 10. | Was statistical analysis appropriate? | Yes |
