# Supplementary material for: mRNA Covid-19 vaccines in pregnancy: A systematic review
Source: PLoS One. 2022 Feb 2;17(2):e0261350. doi: 10.1371/journal.pone.0261350 (PMC8809595; doi:10.1371/journal.pone.0261350)
Supplement: S3 Table — (DOCX) [file pone.0261350.s005.docx]

**S3 Table. Joanna Briggs Institute (JBI) critical appraisal for case report study**

| No | Checklist questions | Gill and Jones, 2021 | Paul and Chad, 2021 |
| --- | --- | --- | --- |
| 1. | Were patient’s demographic characteristics clearly described? | Yes | No |
| 2. | Was the patient’s history clearly described and presented as a timeline? | Yes | Yes |
| 3. | Was the current clinical condition of the patient on presentation clearly described? | Yes | Yes |
| 4. | Were diagnostic tests or assessment methods and the results clearly described? | Yes | Yes |
| 5. | Was the intervention(s) or treatment procedure(s) clearly described? | Yes | Yes |
| 6. | Was the post-intervention clinical condition clearly described? | Yes | Yes |
| 7. | Were adverse events (harms) or unanticipated events identified and described? | Yes | Unclear |
| 8. | Does the case report provide takeaway lessons? | Yes | Yes |
