# Supplementary material for: mRNA Covid-19 vaccines in pregnancy: A systematic review
Source: PLoS One. 2022 Feb 2;17(2):e0261350. doi: 10.1371/journal.pone.0261350 (PMC8809595; doi:10.1371/journal.pone.0261350)
Supplement: S4 Table — (DOCX) [file pone.0261350.s006.docx]

**S4 Table. Joanna Briggs Institute (JBI) critical appraisal for cross-sectional study**

| No | Checklist questions | Kadali et al., 2021 |
| --- | --- | --- |
| 1. | Were the criteria for inclusion in the sample clearly defined? | Yes |
| 2. | Were the study subjects and the setting described in detail? | Unclear |
| 3. | Was the exposure measured in a valid and reliable way? | Yes |
| 4. | Were objective, standard criteria used for measurement of the condition? | Yes |
| 5. | Were confounding factors identified? | No |
| 6. | Were strategies to deal with confounding factors stated? | No |
| 7. | Were the outcomes measured in a valid and reliable way? | Yes |
| 8. | Was appropriate statistical analysis used? | Yes |
