# Supplementary material for: mRNA Covid-19 vaccines in pregnancy: A systematic review
Source: PLoS One. 2022 Feb 2;17(2):e0261350. doi: 10.1371/journal.pone.0261350 (PMC8809595; doi:10.1371/journal.pone.0261350)
Supplement: Attachment — Submitted filename: 4. List of Responses.docx [file pone.0261350.s007.docx]

Title: mRNA Covid-19 Vaccines in Pregnancy: A Systematic Review

**Responses to the reviewer #1 comments:**

We would like to start by thanking the reviewers for careful and constructive comments and questions. It prompted us to change some of the aspects of our original manuscript, mainly the introduction and discussion along with the conclusion. We strongly believe that our manuscript is much better now than it was before the rewriting.

After careful reading of the comments, we decided that many parts of the article definitely need improvements, especially those with grammatical errors and wrong use of tenses. Also, the description of abbreviations within the table has been attached below each table. We tried to incorporate all suggestions made by the reviewer in this new version of our manuscript.

We decided to err on the side of caution and fix the language issue of our manuscript this time.

Reviewer #1: GENERAL : There are several grammatical errors and wrong use of tenses that make significant portions of the text difficult to understand.

- We have modified the previous manuscript into the revised manuscript with the more appropriate writing English this time. We also got a proofread certificate from a language editing center (Enago). We hope that this at least partially addresses the reviewer's concerns about some issues in our manuscript.

ABSTRACT: Page 2, under methods, line 38 EU should be written in full 1st before abbreviating. Throughout the text abbreviations should 1st be written in full. In lines 39-40 what do the authors mean by he databases were queried? Lines 61-62 should be recasted. Grammatical errors and wrong use of tenses.

- We have replaced European Union instead of the EU in the abstract.
- The word queried here means that we conducted the search in the databases mentioned in the abstract. To make it clearer, we have changed the word ‘queried’ into ‘systematically search’.
- We have recasted this sentence. If you think it is still not suitable, please any feedbacks are welcomed.

INTRODUCTION: Page 3,line 69, which pandemic is being referred to? The statement in lines 74-75 about the risk of severe COVID 19 in pregnancy seems to be at variance with the 1st sentence in the objective section of the abstract. The term "pregnant women should be substituted for pregnant people throughout the text. The aim of the study in page 3 lines 87-90 should be recasted.

- The term pandemic refers to the Covid-19 pandemic and we have changed ‘Covid-19 pandemic’ in this revision.
- We agree with the reviewer which this sentence seems to be at variance with the first sentence in the objective. Since pregnancy has been known to be a factor that leads to poorer outcomes in Covid-19 infection, we decided to revise this sentence so that it would be linear to our objective in the abstract.
- Recently, some opinions suggest the use of pregnant people instead of pregnant women. For this reason, we used this term in our previous manuscript. However, we consider changing the term pregnant people throughout the texts as per the reviewer's suggestion in this revised manuscript.
- We have recast the aim and integrated it into the end of the second paragraph.

METHODS: Several grammatical errors. Under inclusion criteria, lines 100-101 page 4, number 4 is no clear. Where studies that were not conducted in English included? Page 5,under statistical analysis, pages 139-140 is not clear.

- Some studies do not report the minimum age of the participants, but we decided to include these articles. As a result, we think that it would be more appropriate to just write pregnant women in the inclusion criteria, without restricting the age.
- Criterion no 4 in the previous manuscript has been modified into a clearer sentence. All studies were reported in English. Therefore, we changed ‘no language restriction’ (at line 110 of the previous manuscript) in order to be in line with the inclusion criteria.
- We initially borrow the term Synthesis Without Meta-analysis (SWiM) from PRISMA guideline, for use in systematic reviews examining the quantitative effects of interventions for which meta-analysis of effect estimates is not possible, or not appropriate, for a least some outcomes. Because this review had already adhered to PRISMA guidelines 2020, we decided to omit the SWiM term and simplify this sentence to make it clearer in the revised manuscript.

RESULTS: Page 5,lines 144 is not clear, Are 4.018 and 200 both all the records that were yielded? Several grammatical errors. Under outcomes, page 6 lines 168-171 is not clear,

- In this revised manuscript, the number of studies obtained from our search has been combined into 4218, instead.
- The vaccine efficacy was represented by the infection rate in which the lower infection rate indicates a higher efficacy. We are trying to emphasize that some studies evaluate the efficacy in a different way, i.e. different grouping. In this revised manuscript, we have attempted to improve the grammatical errors.

DISCUSSION: TOO many grammatical errors and wrong use of tenses.

- We have reworked the discussion to justify the writing style with more appropriate use of grammar and tenses as per the reviewer's suggestion. Any feedbacks are welcome if necessary.
