# Supplementary material for: mRNA Covid-19 vaccines in pregnancy: A systematic review
Source: PLoS One. 2022 Feb 2;17(2):e0261350. doi: 10.1371/journal.pone.0261350 (PMC8809595; doi:10.1371/journal.pone.0261350)
Supplement: Attachment — Submitted filename: 4. Responses to Reviewers new.docx [file pone.0261350.s008.docx]

Title: mRNA Covid-19 Vaccines in Pregnancy: A Systematic Review

**Responses to the reviewer #1 comments:**

We would like to start by thanking the reviewers for careful and constructive comments and questions. We strongly believe that our manuscript is much better now than it was before the rewriting.

Reviewer #1:

METHODS: Line 83should be --maternal antibody titre instead of maternal titre antibodies. Likewise, line 85 should be cord blood antigen titre.

- Thanks for the suggestion. We have incorporated your suggestion into our manuscript.

Under search strategy87, it is not clear if all the studies used for this research were conducted between June 20th and 22nd 2020--line 90-91.

- We really appreciate this very useful feedback. We actually performed the search between 20^th^ and 22^nd^ of June, 2021 and include all articles before these date. We have fixed the sentence and we hope the meaning will be clearer after this.

Lines 142-143 is not clear, was the median age of the women actually estimated in weeks?

- Thank you for addressing this issue. We actually mean it as gestational age, not age of the mother. Therefore, we use ‘gestational age’ this time.

Lines 178-179 is not clear.

- Thank you for the insightful feedback. We have paraphrased this sentence, thus it should have a clearer meaning.

DISCUSSION: The following should be written in full 1st--CDC, ICD, Tdap, DENV, RCTS.

- Thank you for the meticulous observation. We have corrected this issue and all of these abbreviations have been written in full at 1st.

Lines 324-326 is not clear, did the authors actually assess pregnancy, delivery and neonatal outcomes in non pregnant women?

- Thank you for spotting this mistake. We realized that we have mistaken to write the comparison as pregnant vs non-pregnant. Indeed, the reference evaluated the outcomes between vaccinated and unvaccinated pregnant women. Additionally, since we have defined pregnancy and delivery outcomes as maternal outcomes, we simplify the outcomes to be maternal outcomes and neonatal outcomes, instead of pregnancy, delivery and neonatal outcomes.
